# The Long-Term Recovery of Parotid Glands in Nasopharyngeal Carcinoma Treated by Intensity-Modulated Radiotherapy

**DOI:** 10.3389/fonc.2021.665837

**Published:** 2021-05-07

**Authors:** Shun Tasaka, Keiichi Jingu, Noriyoshi Takahashi, Rei Umezawa, Takaya Yamamoto, Yojiro Ishikawa, Kazuya Takeda, Yu Suzuki, Noriyuki Kadoya

**Affiliations:** Department of Radiation Oncology, Tohoku University Graduate School of Medicine, Sendai, Japan

**Keywords:** radiotherapy, parotid, IMRT, QOL, xerostomia

## Abstract

**Background:**

Xerostomia is one of the most common adverse events of radiotherapy in head and neck cancer patients. There have been many reports on functional changes of the parotid gland after radiation therapy, but there have been few reports on the volume of the parotid gland and its relationship with oral quality of life (QOL) and even fewer reports on longitudinal change of the parotid gland volume. The purpose of this study was to evaluate the long-term change of the parotid gland volume after intensity-modulated radiotherapy (IMRT) for nasopharyngeal carcinoma and the relationship between parotid irradiation dose and xerostomia symptoms.

**Methods:**

We retrospectively analyzed 26 patients with nasopharyngeal cancer treated by IMRT. Longitudinal changes of parotid gland volumes after IMRT were evaluated on CT images. The parotid gland volumes in each period were converted to the ratio to parotid gland volumes before radiotherapy (relative parotid volume). Dunnett’s test was used to evaluate the longitudinal changes in relative parotid volumes at 0-6, 7-18, 19-30, 31-42, 43-54 and 55-66 months after IMRT. We assessed xerostomia 3 years or more after IMRT by measuring the degree of oral moisture using a moisture-checking device (Mucus, Life Co., Ltd.) and oral QOL evaluation by GOHAI (General Oral Health Assessment Index).

**Results:**

The relative parotid volumes during radiotherapy and at 0-6, 7-18, 19-30, 31-42, 43-54 and 55-66 months after IMRT were 75.2 ± 14.3%, 67.2 ± 11.4%, 68.5 ± 15.9%, 72.4 ± 14.8%, 73.0 ± 13.8%, 76.2 ± 17.5%, and 77.1% ± 17.3%, respectively. The parotid volume had recovered significantly at 43-54 and 55-66 months after IMRT, especially in parotids receiving less than 40 Gy as the mean dose. The mean irradiated dose for bilateral parotids showed negative correlations with oral QOL score and oral moisture after a long period.

**Conclusions:**

The parotid volume recovered gradually but had not reached a plateau even 3 years after radiotherapy, especially in parotids receiving less than 40 Gy as the mean dose.

## Introduction

Xerostomia is one of the most common adverse events of radiotherapy in head and neck cancer patients (1). Salivary glands are highly sensitive to radiation and they can be damaged by radiation, leading to xerostomia, which causes a decrease in patients’ quality of life after radiotherapy (2). The mechanism of radiation-induced damage of salivary glands, which has been mainly studied in animal models, is thought to be selective damage of the plasma membrane of secretory cells immediately after radiation exposure, followed by damage of DNA, death of acinar progenitor cells and finally lysis of acinar cells (3–6). Peng et al. suggested that senescence in the salivary gland stem/progenitor cell niche contributes to radiation-induced hyposalivation (7). It is known that the parotid gland can be affected by even 10-15 Gy in conventional fractional radiotherapy (8, 9), and although recovery of parotid gland function is possible with the lapse of time after irradiation with 40-50 Gy, higher doses cause irreversible and permanent xerostomia (10–13). To maintain parotid gland function, the recommended mean irradiated dose for the parotid gland is less than 25-30 Gy, and it was reported that saliva production recovers to the same level as that before treatment (14, 15). Hey et al. observed saliva flow for 36 months after radiation therapy in patients with head and neck cancer and reported that most of the recovery processes were considered complete within that period (16). In a multicenter randomized study (PASSRORT trial), the clinical outcomes of intensity-modulated radiotherapy (IMRT) and 3D-conformal radiotherapy (3D-CRT) in terms of parotid sparing were investigated. It was shown in that study that 39% of the IMRT patients and 74% of the 3D-CRT patients suffered from xerostomia at 12 months after treatment (11). There have been many reports on functional changes of the parotid gland after radiation therapy, but there has been no report on the volume of the parotid gland and its relationship with oral quality of life (QOL) and there are few reports on long-term longitudinal change of the parotid gland volume.

The purposes of this study were to evaluate the long-term longitudinal volume change of the parotid gland after definitive IMRT in patients with nasopharyngeal cancer and to investigate the relationships among irradiated dose for the parotid gland, parotid gland volume and xerostomia symptoms.

## Materials and Methods

This research was carried out in compliance with the content of the Declaration of Helsinki, and it was approved by the institutional review board in our institution.

### Selection of Patients

We retrospectively analyzed consecutive patients with nasopharyngeal cancer who were treated by definitive IMRT combined with chemotherapy in our hospital between 2009 and 2017. Eligibility criteria were as follows: (a) pathologically proven nasopharyngeal carcinoma, (b) longitudinal and regular evaluation by computed tomography (CT) in our hospital and availability of longitudinal data for 3 years or more, (c) no previous surgery for the head and neck area, (d) no history of diseases causing a salivary secretion disorder such as Sjogren’s syndrome, (e) treatment by definitive chemoradiotherapy, and (f) treatment by IMRT.

### Treatment

#### Radiotherapy

All patients received irradiation to bilateral prophylactic cervical lymph node regions in addition to the primary tumor and region of lymph node metastasis by IMRT. Clinical target volume (CTV) 1 was defined as the primary tumor and region of lymph node metastasis with an appropriate margin, and CTV 2 was assigned to bilateral prophylactic cervical lymph node regions. Planning target volume (PTV) was set with a margin of 5 mm to the CTV. Delineation for all patients was done out by single radiation oncologist. PTV 1 was irradiated with 70 Gy in 35 fractions (fr.) and PTV 2 was irradiated with 40-50 Gy in 20-25 fr. using a 6-15 MV X-ray linear accelerator. Re-planning was conducted in the third or fourth week of IMRT for all patients.

#### Chemotherapy

All patients received three cycles of cisplatin (100 mg/m^2^/day) during radiotherapy. In advanced cases, adjuvant chemotherapy consisting of cisplatin and 5-flurouracil was performed after concurrent chemoradiotherapy.

#### Parotid Gland Volumes

Longitudinal head-and-neck CT data were transferred to a 3D-radiation therapy planning system (Eclipse, Varian Medical System Inc.) using the digital imaging and communication in medical format (DICOM). For longitudinal evaluation of parotid gland volumes, the CT scans were categorized into 7 periods: during RT and periods 0, 1, 2, 3, 4 and 5, representing the timing of CT scans at re-planning, 0-6 months, 7-18 months, 19-30 months, 31-42 months, 43-54 months, and 55-66 months after completion of IMRT. A CT scan with a slice thickness of 2 mm or less was performed with contrast medium after at least 4 hours of fasting. Two radiation oncologists with 5 years and 15 years of clinical experience re-contoured the parotid glands on a three-dimensional radiation therapy planning system and measured and recorded the parotid gland volumes.

### Measurement of the Degree of Moisture in the Mouth and QOL Evaluation

We assessed xerostomia 3 years or more after IMRT by measuring the degree of moisture in the mouth using a moisture-checking device (Mucus, Life Co., Ltd.) and oral QOL evaluation by GOHAI (General Oral Health Assessment Index) (17).

The degree of moisture in the mouth was measured 3 times continuously by placing the moisture-checking device at the center of the tongue, and the average value of the 3 measurements was used as the measured value. Patients were instructed not to eat, drink or gargle for at least 30 minutes before the measurements and to rest for at least 5 minutes before the measurements. Measured values of the moisture-checking device were defined as follows according to a previous report (18): <25, advanced drying; 25-28, moderate drying; 28-30, mild drying; 30 <, normal.

The GOHAI questionnaire is a questionnaire about the frequency of problems caused by a bad condition in the mouth during the past 3 months and consists of 12 questions for eating, speaking, pain, discomfort, worry, and social functioning. Five scales (l = always, 2 = often, 3 = sometimes, 4 = seldom, and 5 = never) are used for answers and QOL is evaluated by the total score of 12 questions (with the lowest total score 12 and highest total score 60 and a higher total score indicating higher quality of life) (18).

### Statistical Analysis

Statistical analyses were performed with SPSS 26.0. Since there were considerable individual differences in parotid gland volumes, the parotid gland volumes in each period were converted to the ratio to parotid gland volumes before radiotherapy (relative parotid volume). Dunnett’s test was used to evaluate the longitudinal changes in relative parotid volumes for periods 0-5. Using Student’s t-test, we compared the relative parotid volumes in each period for patients receiving less than 40 Gy with those for patients receiving 40 Gy or more, those for patients aged less than 50 years with those for patients aged 50 years or older and those for patients who had a smoking history with those for patients who had no smoking history. Correlation analysis was performed for mean irradiated dose of the bilateral parotids, relative bilateral parotid volumes, measured values of the moisture-checking device and GOHAI score at the last observation date. For all analyses, p <0.05 was considered statistically significant.

## Results

### Eligible Patients’ Characteristics

Twenty-six patients were enrolled in this study [18 males and 8 females; median age, 51.0 years (range, 20-77 years)]. In the T classification, 9 cases were T1, 10 cases were T2, 3 cases were T3, and 4 cases were T4, and in the N classification, 4 cases were N0, 14 cases were N1, 6 cases were N2, and 2 cases were N3. Two cases were Stage I, 13 cases were Stage II, 6 cases were Stage III, and 5 cases were Stage IV (classified according to UICC TNM classification 7th edition). All patients received platinum-based chemotherapy in combination with IMRT concurrently. The mean irradiated dose ± SD of 52 parotid glands in 26 patients was 42.2 ± 11.5 Gy. The patients’ characteristics are shown in **Table 1**.

**Table 1:** Patients' characteristics.

		total number = 26
Gender	male	18 (69.2%
	female	8 (30.8%)
Age		51 y-o (range, 20-77)
T stage	T1	9 (34.6%)
	T2	10 (38.5%)
	T3	3 (11.5%)
	T4	3 (11.5%)
N stage	N0	4 (15.4%)
	N1	14 (53.8%)
	N2	6 (23.1%)
	N3	2 (7.7%)
Stage	1	2 (7.7%)
	2	13 (50%)
	3	6 (23.1%)
	4	5 (15.4%)
Smoking	never	13 (50%)
	missing	11 (42.3%)
	ever	2 (7.7%)
Bilateral parotids volume before RT	Mean SD	61.9 cm^3^ 15.4 cm^3^
Mean dose for bilateral parotids	Mean SD	41.2 Gy 10.2 Gy

### Parotid Volumes

**Figure 1:**
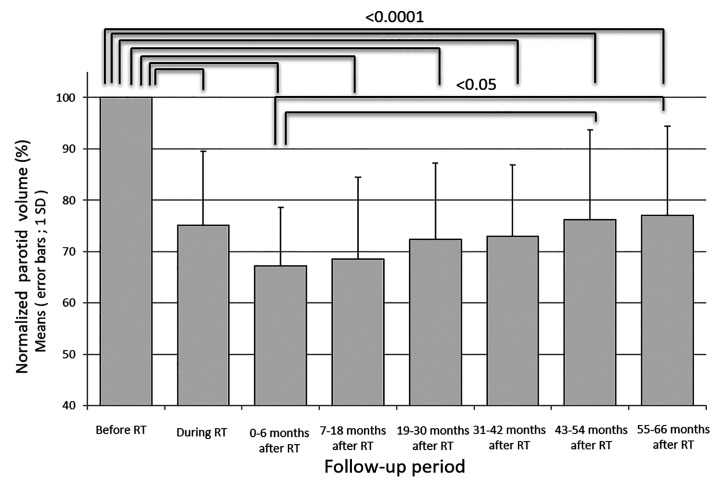
Longitudinal changes in relative parotid volumes during and after radiotherapy.

**Figure 2:**
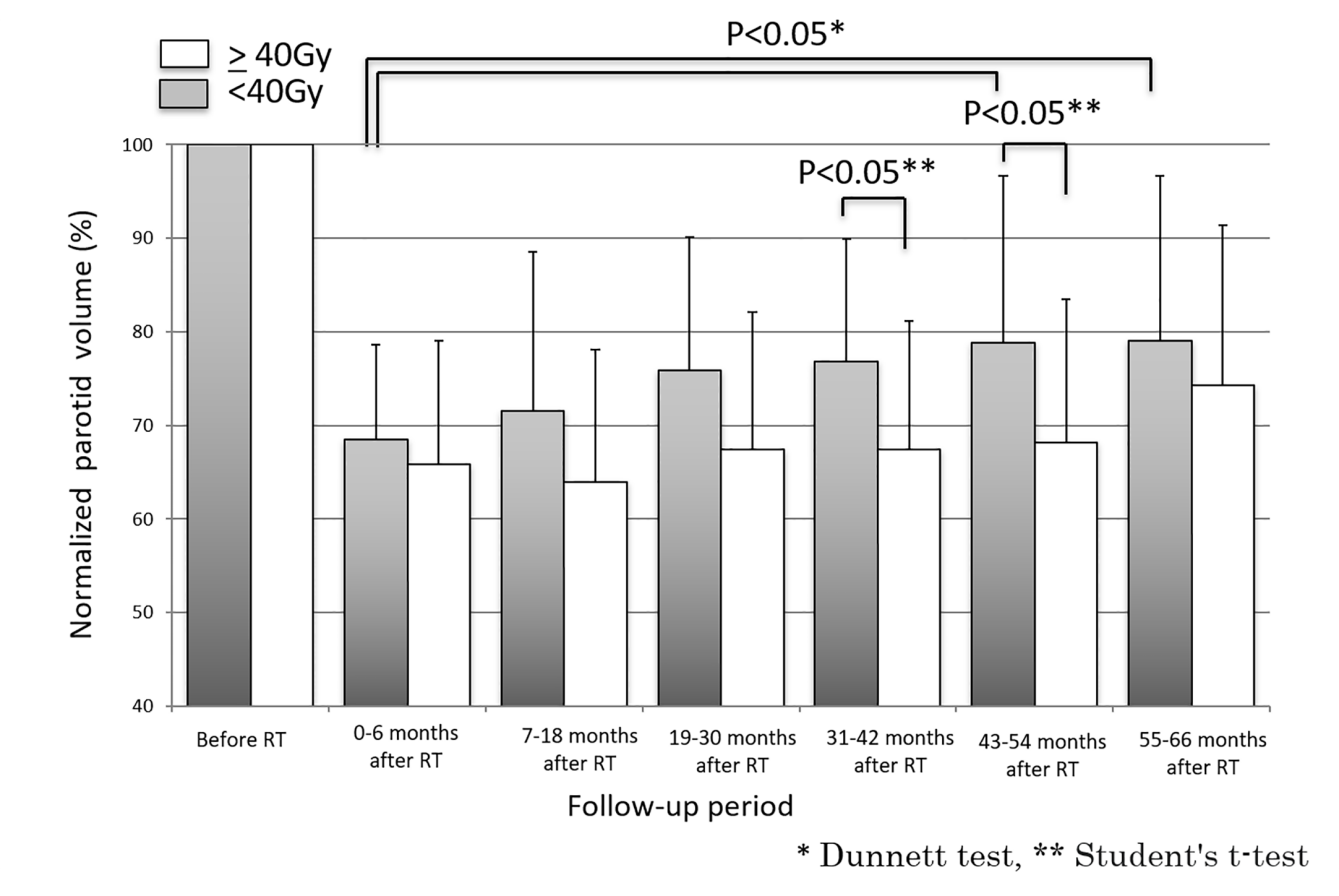
Longitudinal changes in relative parotid volumes after radiotherapy for parotids irradiated with less than 40 Gy and for parotids irradiated with 40 Gy or more.

**Figure 3:**
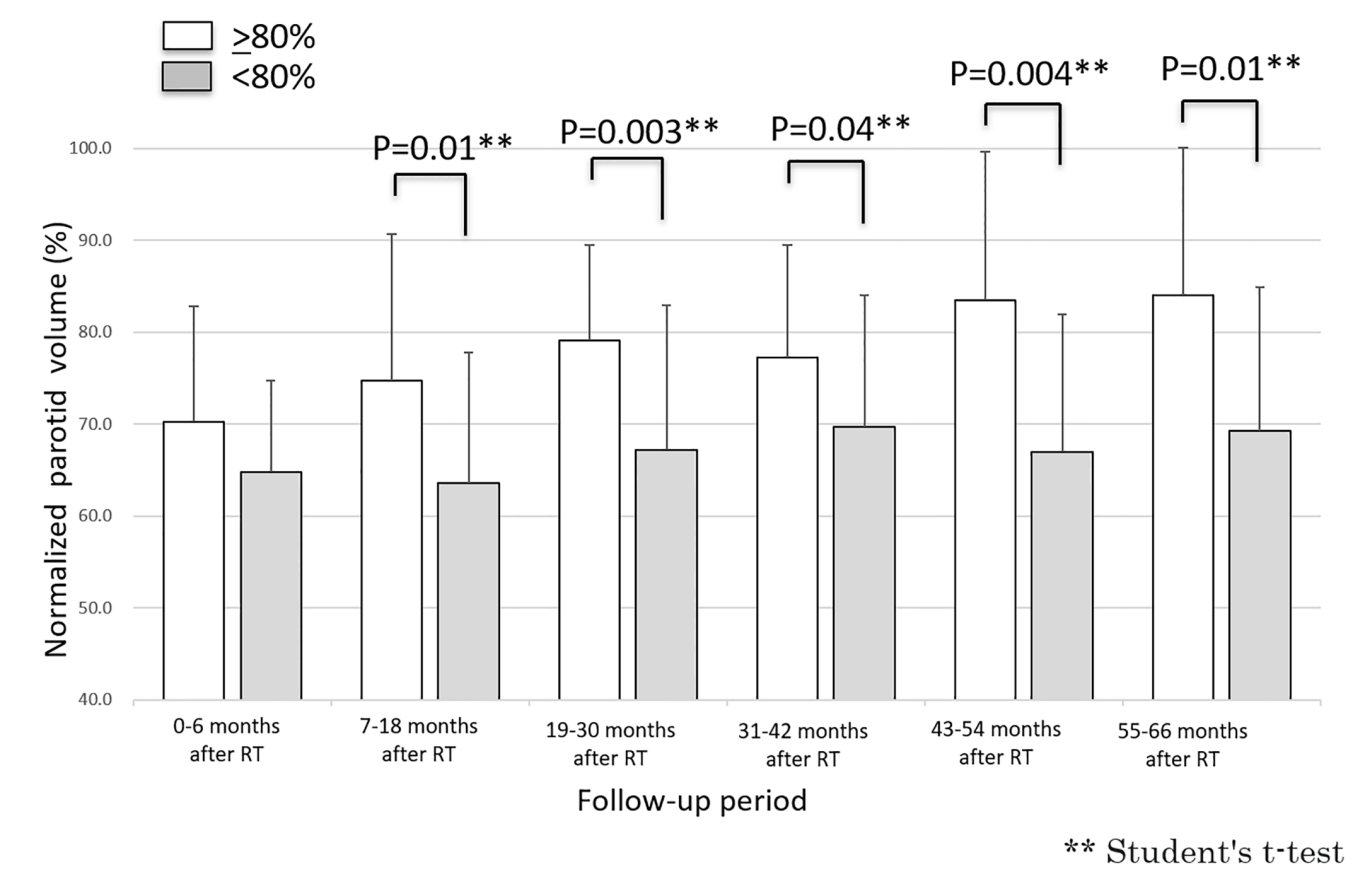
Longitudinal changes in relative parotid volumes after radiotherapy for parotid with volumes of 80% or more at re-planning and those for parotids with volumes of less than 80% at re-planning.

Fifty-two parotid glands in 26 patients were analyzed. The parotid volume before radiotherapy was 31.1 ± 7.7 cm^3^ (mean ± SD). The relative parotid volumes at re-planning and periods 0, 1, 2, 3, 4 and 5 were 75.2 ± 14.3% (n = 52), 67.2 ± 11.4% (n = 52), 68.5 ± 15.9% (n = 52), 72.4 ± 14.8% (n = 52), 76.2 ± 17.5% (n = 52), 76.2 ± 17.5% (n = 34), and 77.1% ± 17.3% (n = 32), respectively (**Figure 1**). The relative parotid volumes were significantly shrunken by radiotherapy (Dunnett’s test: p<0.0001 for all periods). The nadir of the relative parotid volume appeared in period 0 and relative parotid volume had recovered significantly in periods 4 and 5 (Dunnett’s test: p=0.015 and p=0.008, respectively). Although the differences were not statistically significant, periods 1, 2 and 3 also showed gradual recovery of relative parotid volume from period 0. The relative volumes of parotids that were irradiated with less than 40 Gy as the mean dose in periods 0, 1, 2, 3, 4 and 5 were 68.8 ± 10.1%, 71.5 ± 16.6%, 75.5 ± 14.2%, 76.5 ± 12.9%, 79.1 ± 17.9% and 79.9 ± 17.7%, respectively (**Figure 2**). The relative volumes of parotids that were irradiated with less than 40 Gy as the mean dose had recovered significantly in periods 4 and 5 (Dunnett’s test: p=0.036 and p=0.035, respectively). The relative volumes of parotids that were irradiated with 40 Gy or more as the mean dose in periods 0, 1, 2, 3, 4 and 5 were 64.9 ± 13.0%, 64.0 ± 14.1%, 67.9 ± 14.8%, 67.8 ± 13.8%, 72.2 ± 16.6% and 72.4 ± 16.3%, respectively (**Figure 2**). The relative volumes of parotids with 40 Gy or more as the mean dose had not recovered significantly. In periods 3 and 4, the relative volumes of parotids irradiated with less than 40 Gy were significantly larger than those of parotids irradiated with 40 Gy or more (Student’s t-test: p=0.014 and p=0.034, respectively). The relative parotid volumes in patients whose relative parotid volume at re-planning was 80% or more were significantly larger in all periods, except for period 0 than those in patients whose relative parotid volume at re-planning was less than 80% (Student’s t-test: p=0.08, p=0.01, p=0.003, p=0.04, p=0.004 and p=0.01, respectively) (**Figure 3**). There was no significant difference between the relative volumes of parotids in patients aged less than 50 years and those in patients aged 50 years or older in any of the periods (Student’s t-test: p=0.87, p=0.79, p=0.62, p=0.99, p=0.96 and p=0.83, respectively). There was no significant difference between the relative volumes of parotids in patients who had a smoking history and those in patients who had no smoking history in any of the periods (Student’s t-test: p=0.59, p=0.25, p=0.58, p=0.67, p=0.66 and p=0.68, respectively).

### Oral Moisture and QOL Score

**Figure 4:**
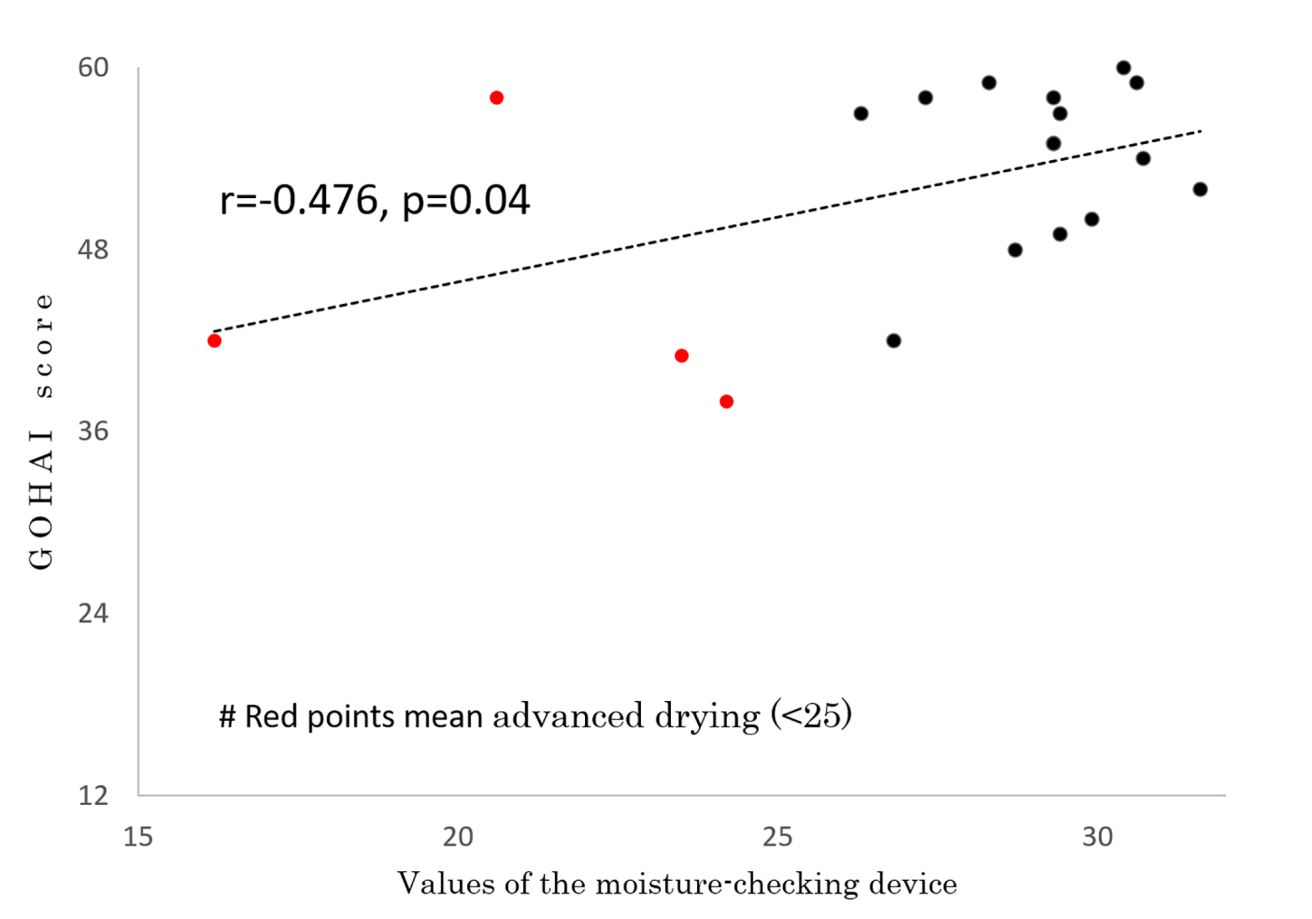
Correlation between GOHAI score and values of the moisture-checking device.

**Figure 5:**
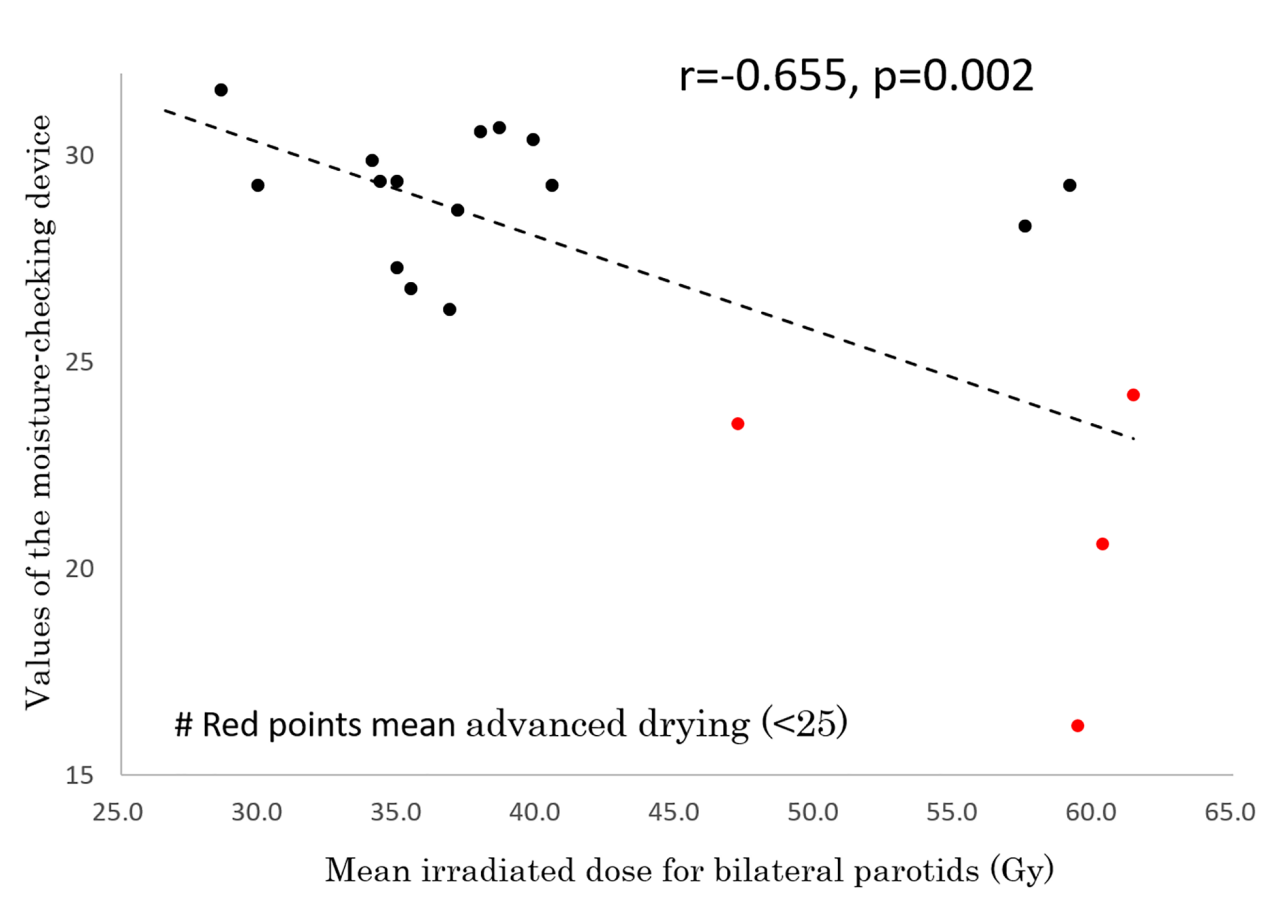
Correlation between mean irradiated dose for bilateral parotids and values of the moisture-checking device.

In 19 of the 26 patients, the degree of moisture in the mouth was measured by a moisture-checking device and oral QOL was assessed by GOHAI at 59.2 ± 7.2 months (range, 43-66 months) after IMRT. No patients had used pilocarpine for at least 6 months before those examinations. Measurements could not be performed in the other 7 patients because they did not visit our hospital or their follow-up period did not reach period 4 or 5. The mean measured value of the moisture-checking device was 27.5 ± 3.9 (range, 16.2 - 30.7). The mean GOHAI score was 52.2 ± 7.0 (range, 42 - 60). A correlation was found between GOHAI score and values of the moisture-checking device (r=0.476, p=0.04) (**Figure 4**). A negative correlation was found between mean irradiated dose for bilateral parotids and values of the moisture-checking device (r=-0.655, p=0.002) (**Figure 5**). No significant correlation was found between mean irradiated dose for bilateral parotids and GOHAI score (r=-0.248, p=0.31). No significant correlation was found between relative volumes of bilateral parotids and measured values of the moisture-checking device (r=0.193, p=0.443). There was also no significant correlation between relative volumes of bilateral parotids and GOHAI score (r=0.067, p=0.793). There was no significant difference in measured values of the moisture-checking device or GOHAI score between patients aged less than 50 years and patients aged 50 years or older in any of the periods (Student’s t-test: p=0.96 and p=0.31, respectively).

## Discussion

Although the mechanism of radiation-induced decrease in parotid volumes has not been elucidated, it was reported that the decrease in parotid volumes might be due to the loss of acinar cells or fibrosis and that the recovery of parotid volumes might be due to regeneration of acinar cells (5, 19, 20). In this study, longitudinal changes in parotid gland volume were analyzed in patients with nasopharyngeal carcinoma who were treated by IMRT with chemotherapy, and the relationships of mean irradiated dose for the parotid with parotid gland volume and xerostomia symptoms were assessed. It was found that the parotid volume decreased rapidly during and immediately after radiotherapy and then recovered gradually but had not reached a plateau even 3 years after radiotherapy. There have been few reports on long-term changes in the parotid after radiotherapy for head and neck cancer. The present study is the longest-term study in which changes in parotid volume after radiotherapy for patients with head and neck cancer were investigated. To the best of our knowledge, there have only been two reports on long-term changes in parotid volume after radiotherapy for head and neck cancer. Tomitaka et al. evaluated parotid volumes at 2 weeks and 6, 12, and 24 months after radiotherapy in 15 patients who received 30 Gy/15 fr. as preoperative radiotherapy with opposed lateral fields for advanced oral cancer, and they reported that the parotid volumes decreased, reached a nadir at 6 months after radiotherapy, and had recovered at 24 months after radiotherapy (21). Hey et al. reported that most of the recovery process from radiation-induced salivary gland injury was completed within 36 months after radiotherapy (16), and their results are almost consistent with the results of our study. However, our data for parotid volumes 3 years and more after radiotherapy suggest that, even after that, further recovery can be expected, especially in parotids irradiated with 40 Gy or less as the mean dose. Furthermore, the relative volumes of parotids receiving less than 40 Gy tended to be larger than those of parotids receiving more than 40 Gy in any of the periods except for immediately after IMRT, especially after a long period. Our data also showed that there is a negative correlation between objective xerostomia symptoms after a long period and mean irradiated dose for bilateral parotids. This suggests that reducing the mean irradiated dose for the parotid gland as much as possible leads to long-term improvement of the oral condition. However, since it could not be analyzed in this study because of small number of patients and since there were no data for patients whose contralateral parotids were irradiated with less than 26 Gy, which has often been recommended (22, 23), as the mean dose, it is unclear whether further recovery of parotid volume and oral QOL can be expected if the mean dose for the contralateral parotid is 26 Gy or less.We also showed that there was a weak correlation between oral QOL score and measured values of the moisture-checking device. This might be because there were only 6 patients whose bilateral parotids were irradiated with more than 40 Gy as a mean dose and it might be because the GOHAI score did not show only wetness in the oral cavity. Toxicities after radiotherapy for head and neck cancers are intricately intertwined (24). Therefore, it is reasonable that there was no significant correlation between oral QOL score and mean irradiated dose for bilateral parotid glands in this study. Furthermore, it is known that patient-rated and physician-rated toxicities for xerostomia are not matched completely (25). It is difficult to evaluate xerostomia accurately.

There are few reports of GOHAI being used in the field of radiotherapy. The importance of not only outcome evaluation by medical staff but also subjective evaluation by patients themselves has been recognized, but the European Organisation for Research and Treatment of Cancer Quality of Life Questionnaire Head and Neck Module (EORTC QLQ-HN35), which is the most commonly used questionnaire for patients treated by radiotherapy for head and neck cancers, is difficult to answer because it consists of many questions. The GOHAI questionnaire consists of easy questions and even elderly patients can complete it in about 5 minutes and it is therefore not a burden for patients.

This study had some limitations. The number of eligible patients was only 26 and the number of patients in whom measurements by a moisture-checking device and evaluation of oral QOL were performed was only 19. Due to the small number of cases, there might have been some factors that were not clarified in the analysis. Since this study was a retrospective study and was performed only for patients for whom long-term follow-up was possible, the possibility of bias in selection of patients cannot be ruled out. In addition, although only parotid volumes were evaluated in this study, the irradiation field also included the submandibular gland, sublingual gland, and other salivary glands, and radiation-induced damage of salivary glands cannot be neglected for oral moisture or QOL. However, total saliva is mainly produced by the parotid gland, and radiation-induced injury to salivary glands mainly results from damage to parotid glands (1). Therefore, we evaluated only parotid volumes in this study. Furthermore, we did not consider fatty change of the parotid after radiotherapy in this study.

In conclusion, parotid volume continued to recover gradually even 3 years after radiotherapy, especially for parotids receiving less than 40 Gy as the mean dose.

## Data Availability Statement

The raw data supporting the conclusions of this article will be made available by the authors, without undue reservation.

## Ethics Statement

The studies involving human participants were reviewed and approved by Ethics Committee of Tohoku University Graduate School of Medicine. Written informed consent for participation was not required for this study in accordance with the national legislation and the institutional requirements.

## Author Contributions

Conception and design or analysis and interpretation of data: ST, KJ, NT, TY, YI, KT, and YS. Drafting of the manuscript or revising it for important intellectual content: ST, KJ and NK. Final approval of the version to be published: ST, KJ, and NT. All authors contributed to the article and approved the submitted version.

## Conflict of Interest

The authors declare that the research was conducted in the absence of any commercial or financial relationships that could be construed as a potential conflict of interest.

## Abbreviations

IMRT, intensity-modulated radiotherapy; 3D-CRT, 3D-conformal radiotherapy; QOL, quality of life; CTV, clinical target volume; PTV, planning target volume; GOHAI, General Oral Health Assessment Index.
